# Birth-and-Death Evolution of the Fatty Acyl-CoA Reductase (FAR) Gene Family and Diversification of Cuticular Hydrocarbon Synthesis in *Drosophila*

**DOI:** 10.1093/gbe/evz094

**Published:** 2019-05-10

**Authors:** Cédric Finet, Kailey Slavik, Jian Pu, Sean B Carroll, Henry Chung

**Affiliations:** 1 Université de Lyon, Institut de Génomique Fonctionnelle de Lyon, CNRS UMR 5242, École Normale Supérieure de Lyon, Université Claude Bernard Lyon 1, France; 2 Howard Hughes Medical Institute and Laboratory of Molecular Biology, University of Wisconsin, Madison; 3 Department of Entomology, Michigan State University; 4 Ecology, Evolutionary Biology and Behavior, Michigan State University; 5 Department of Biology, University of Maryland, College Park, MD; 6 PhD Program in Virology, Division of Medical Sciences, Harvard University, Boston, MA, USA

**Keywords:** birth-and-death evolution, cuticular hydrocarbons, *Drosophila*, fatty acyl-CoA reductase, oenocytes

## Abstract

The birth-and-death evolutionary model proposes that some members of a multigene family are phylogenetically stable and persist as a single copy over time, whereas other members are phylogenetically unstable and undergo frequent duplication and loss. Functional studies suggest that stable genes are likely to encode essential functions, whereas rapidly evolving genes reflect phenotypic differences in traits that diverge rapidly among species. One such class of rapidly diverging traits are insect cuticular hydrocarbons (CHCs), which play dual roles in chemical communications as short-range recognition pheromones as well as protecting the insect from desiccation. Insect CHCs diverge rapidly between related species leading to ecological adaptation and/or reproductive isolation. Because the CHC and essential fatty acid biosynthetic pathways share common genes, we hypothesized that genes involved in the synthesis of CHCs would be evolutionary unstable, whereas those involved in fatty acid-associated essential functions would be evolutionary stable. To test this hypothesis, we investigated the evolutionary history of the fatty acyl-CoA reductases (FARs) gene family that encodes enzymes in CHC synthesis. We compiled a unique data set of 200 FAR proteins across 12 *Drosophila* species. We uncovered a broad diversity in FAR content which is generated by gene duplications, subsequent gene losses, and alternative splicing. We also show that FARs expressed in oenocytes and presumably involved in CHC synthesis are more unstable than FARs from other tissues. Taken together, our study provides empirical evidence that a comparative approach investigating the birth-and-death evolution of gene families can identify candidate genes involved in rapidly diverging traits between species.

## Introduction

Multigene families are important contributors to molecular and organismal evolution. Member genes descend from single founder genes that duplicate, then diverge in sequence (Nei and Rooney 2005). Several models have been proposed to account for how multigene families evolve. For example, the concerted evolution model hypothesizes that all member genes in the family evolve as a unit. This model is capable of explaining aspects of the evolution of clustered ribosomal RNAs (Brown et al. 1972). In contrast, the birth-and-death model proposes that the members of a gene family evolve independently, meaning that while some members of a gene family are phylogenetically stable, others are unstable and are gained or lost over time by DNA duplications, deletions, and other pseudogenization events (Hughes and Nei 1989; Lynch and Conery 2000; Sjodin et al. 2007; Plata and Vitkup 2014). Gene repertoire expansion and contraction has been found in diverse gene families such as innate immune genes (Zhang et al. 2015; Sackton et al. 2017), plant secondary metabolic genes (Lespinet et al. 2002; Jiang et al. 2015; Wang et al. 2018), developmental transcription factors (Amores et al. 2004; Tanabe et al. 2005; Finet et al. 2013), and snake toxin genes (Dowell et al. 2016). The accumulated evidence indicates that most gene families evolve according to the birth-and-death model.

It has been suggested that gene birth-and-death could provide insights into the origins of phenotypic novelties (Nei and Rooney 2005; Benton 2015). One example is the cytochrome P450 multigene family (Feyereisen 1999). In animals, all phylogenetically stable P450s encode enzymes that have known endogenous substrates, whereas most of the unstable P450s encode enzymes that play roles in xenobiotic detoxification (Thomas 2007; Chung et al. 2009; Good et al. 2014). Such observations suggest that members of a gene family with core functions in development and physiology are unlikely to be gained or lost during evolution, whereas members with rapidly evolving functions between species, such as environmental toxin detoxification, would be gained and lost as species adapt to different habitats (Tatusov et al. 1997; Rubin et al. 2000; Hahn et al. 2007; Thomas 2007; Guo 2013).

Frequent gain and loss of members of a gene family is also apparent in the evolution of the gene families involved in insect chemoreception, such as the gustatory receptor, odorant receptor, and odorant-binding protein gene families, which have been shown to expand and contract by birth-and-death evolution (Vieira et al. 2007; Benton 2015). For instance, in the *Drosophilamelanogaster* group, odorant-binding protein content has evolved more rapidly in the specialist lineages than in their closest generalist relatives (Vieira et al. 2007). Gustatory receptor and odorant receptor repertoires have also been shown to differ considerably between species, with host specialist species losing genes at a much faster rate than their closest generalist sibling species (McBride 2007; McBride et al. 2007). Together, these data suggest that the gene families involved in chemoreception experience rapid evolution among species, and that ecological diversification and natural selection may play major roles in this process.

Among chemoreceptor ligands in insects, short-range or contact pheromones are chemicals that constitute the major signal used in mate recognition between two individuals (Yew and Chung 2015). In many insects, these pheromone components are cuticular hydrocarbons (CHCs) (Howard and Blomquist 2005). Composed of alkanes, methyl-branched alkanes, and unsaturated hydrocarbons, CHCs form a waxy layer on the cuticle of the insect, where their primary role is probably in maintaining water balance, preventing desiccation due to cuticular water loss (Gibbs 1998). Because of the dual roles that insect CHCs play in both ecological adaptation and chemical signaling, these compounds can evolve rapidly among species adapted to living in different environments and habitats (Jallon and David 1987; Chung and Carroll 2015).

**Fig. 1.**
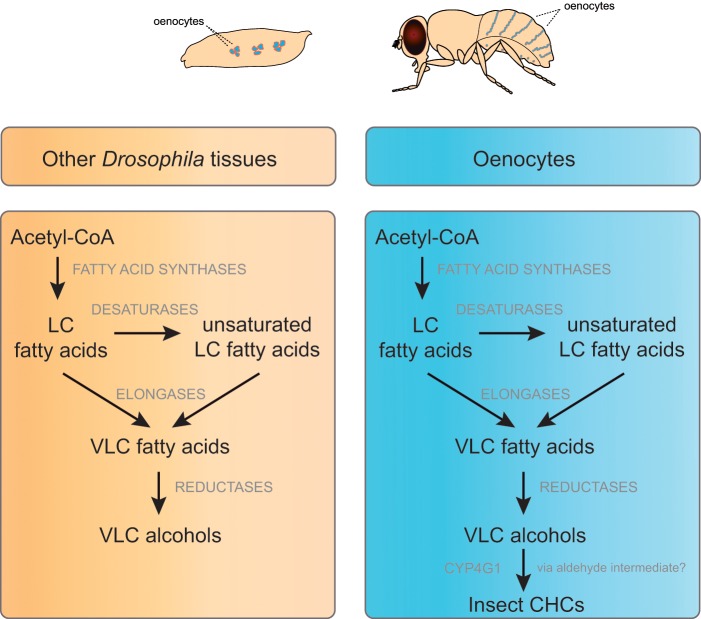
—Fatty acid biosynthesis pathway in *Drosophila*. This stepwise process takes place in many different cell types and is catalyzed by several classes of cell-type-specific enzymes that generate the diversity of fatty acids found in the organism. Some of these fatty acids are reduced to alcohols by specific reductases. In oenocytes, an additional step consists of the conversion of some of the very long chained (VLC) alcohols to hydrocarbons by a single cytochrome P450, CYP4G1, which exists in all insect genomes sequenced to date.

The mechanisms underlying the rapid evolution of CHC content are not well understood. The diversity of insect CHCs is shaped by the action of several families of enzymes in specialized cells called oenocytes that are located beneath the insect cuticle (Billeter et al. 2009). These gene families include fatty acid synthases, desaturases, elongases, and reductases, which make up the ubiquitous fatty acid synthesis pathway in almost all cells. In the oenocytes, a single decarbonylase, *Cyp4g1*, converts some of the products of this pathway into CHCs in *Drosophila* (Qiu et al. 2012) (fig. 1). Orthologs of *Cyp4g1* has been found in multiple species of insects and have been shown to perform similar functions (Chen et al. 2016; Yu et al. 2016; MacLean et al. 2018). Only a handful of genes encoding enzymes involved in the biosynthesis of CHCs have been identified and characterized so far in *Drosophila* (Chung and Carroll 2015). *mFAS*, which encodes a fatty acyl-CoA synthase expressed in oenocytes, is involved in the production of methyl-branched CHCs (Chung et al. 2014). The desaturases, *desat1* and *desat2*, play a role in the synthesis of hydrocarbons with at least one double bond at the Z-7 and Z-5 positions, respectively (Dallerac et al. 2000; Takahashi et al. 2001), whereas *desatF* catalyzes the formation of a second double bond in dienes (Chertemps et al. 2006; Shirangi et al. 2009). Likewise, *eloF* is the only elongase known to be involved in the female-specific elongation of long-chain dienes in *D. melanogaster* (Chertemps et al. 2007). A recent genome-wide association study identified novel fatty acid biosynthesis pathway enzymes that are associated with intraspecific CHC variation in *D. melanogaster*, including three elongases (*CG30008*, *CG18609*, and *CG9458*) and two fatty acyl-CoA reductases (FARs) (*CG13091* and *CG10097*) (Dembeck et al. 2015).

The majority of the enzymes involved in the synthesis of the CHCs in *Drosophila* are still unknown. The identification of such enzymes has been hampered by experimental difficulties because the CHC pathway has many genes in common with the more pleiotropic fatty acid biosynthesis pathway, and the gene families involved are usually very large (Chung and Carroll 2015). We hypothesized that because CHCs are rapidly evolving traits, the genes underlying their synthesis may be rapidly evolving between closely related species. Here, we tested this hypothesis by focusing on the evolution of the FAR gene family that encodes enzymes catalyzing the reduction of acyl-CoA to alcohols and aldehydes (Riendeau and Meighen 1985; Cinnamon et al. 2016) (fig. 1). We show that the FAR gene family evolves following a birth-and-death model. We took advantage of differential molecular evolutionary features between stable and unstable FARs to identify the FARs that are likely to be involved in core functions (fatty acid biosynthesis) and those likely to be involved in rapidly evolving functions between species (CHC biosynthesis).

## Materials and Methods

### Fly Stocks

The *Canton-S* strain or the *Xout* strain was used as the wild-type *D. melanogaster* strain for *in situ* hybridization. All RNAi lines were obtained from the Vienna *Drosophila* RNAi Center (Dietzl et al. 2007). The tubulin-GAL4 (Bloomington Stock #5138) strain was obtained from the Bloomington *Drosophila* Stock Center. All flies were maintained at room temperature on standard Bloomington recipe *Drosophila* food.

### Data Collection

FAR genes were identified in 12 complete *Drosophila* genomes by TBlastN using *D. melanogaster* sequences as probes. *Drosophila ananassae*, *D. erecta*, *D. grimshawi*, *D. melanogaster*, *D. mojavensis*, *D. persimilis*, *D. pseudoobscura*, *D. sechellia*, *D. simulans*, *D. virilis*, *D. willistoni*, and *D. yakuba* genomes were retrieved from the FlyBase website (http://flybase.org). We experimentally checked the sequence of some FARs by polymerase chain reaction, especially when we started to use early versions of the released genomes. We completed the sequence of the *D. yakuba* transcript *FarO* (*DyakGE28152*) and deposited the new sequence in the EMBL database (accession number LT996250). Sequence alignment and tree files are downloadable from Dryad (doi:10.5061/dryad.s31rc70).

### Phylogenetic Analyses

Amino acid sequences were aligned with MUSCLE (Edgar 2004), manually adjusted, and conserved blocks were used for phylogenetic reconstruction. Maximum-likelihood searches were performed using RAxML 7.3.5 (Stamatakis 2006), which allows efficient maximum-likelihood analyses of large data sets. All searches were completed under the LG substitution matrix with final likelihood evaluation using a gamma distribution. One hundred bootstrap replicates were conducted for support estimation. In addition, we used the PhyloBayes 3.3 program (Lartillot et al. 2009), which implements a GTR + Γ model. We ran two independent chains for at least 21,000 cycles and discarded the first 5,000 cycles as burn-in. Convergence and chain mixing were checked by using within-PhyloBayes tools (bpcomp and tracecomp).

### Detection of Positive Selection

Candidate genes were tested for signatures of positive selection based on the ratio *ω* = *d*_N_/*d*_S_ (nonsynonymous/synonymous substitution rates) using the program codeml of PAML v.4.8 (Yang 2007). The alignment (and associated tree) used as PAML input was not the complete 200-sequence data set, but a subdata set limited to the clade of interest and its closest outgroup. We compared the null model (*ω* fixed to 1) to the alternative branch-site model that allows some sites to have an *ω* > 1 in specified branches (see table 2, first column). The two models were compared using a likelihood ratio test (degrees of freedom = 1 in all our analyses, see table 2). A *P* value <0.05 means that the model with positive selection better explains the data. The Holm–Bonferroni correction was employed to account for the problem of multiple hypothesis test (Anisimova and Yang 2007). The codon alignment, used as input in PAML, was generated using the software PAL2NAL (Suyama et al. 2006).

**Table 2:** Statistics for the Branch-Site Test of Positive Selection

Foreground Branch	Null Model		Alternative Model		−2Δln *L*	df	*P* Value	Holm–Bonferroni Corrected *P* Value
	Parameters	ln *L*	Parameters	ln *L*				
*DanaGF17060*	*p*_0_ = 0.561	−11,225.209	*p*_0_ = 0.744	−11,219.170	12.078	1	5.1 × 10^−4^	2 × 10^−3^
	*p*_1_ = 0.126		*p*_1_ = 0.167					
	*p*_2a_ = 0.255		*p*_2a_ = 0.073					
	*p*_2b_ = 0.058		*p*_2b_ = 0.016					
	*ω*_0_ = 0.124		*ω*_0_ = 0.124					
	*ω*_1_ = 1		*ω*_1_ = 1					
	*ω*_2_ = 1		*ω*_2_ = 11.202					
*DanaGF17063*	*p*_0_ = 0.709	−11,226.499	*p*_0_ = 0.762	−11,223.058	6.884	1	8.7 × 10^−3^	1.7 × 10^−2^
	*p*_1_ = 0.167		*p*_1_ = 0.174					
	*p*_2a_ = 0.1		*p*_2a_ = 0.051					
	*p*_2b_ = 0.024		*p*_2b_ = 0.012					
	*ω*_0_ = 0.123		*ω*_0_ = 0.126					
	*ω*_1_ = 1		*ω*_1_ = 1					
	*ω*_2_ = 1		*ω*_2_ = 14.503					
*DsecGM26015*	*p*_0_ = 0.378	−4,554.441	*p*_0_ = 0.800	−4,529.358	50.167	1	1.4 × 10^−12^	8.4 × 10^−12^
	*p*_1_ = 0.063		*p*_1_ = 0.146					
	*p*_2a_ = 0.479		*p*_2a_ = 0.045					
	*p*_2b_ = 0.080		*p*_2b_ = 0.009					
	*ω*_0_ = 0.116		*ω*_0_ = 0.117					
	*ω*_1_ = 1		*ω*_1_ = 1					
	*ω*_2_ = 1		*ω*_2_ = 257.742					
*DperGL27182/DpseGA32357*	*p*_0_ = 0.766	−4,569.562	*p*_0_ = 0.815	−4,565.092	8.941	1	2.8 × 10^−3^	8.4 × 10^−3^
	*p*_1_ = 0.136		*p*_1_ = 0.147					
	*p*_2a_ = 0.083		*p*_2a_ = 0.032					
	*p*_2b_ = 0.015		*p*_2b_ = 0.006					
	*ω*_0_ = 0.125		*ω*_0_ = 0.125					
	*ω*_1_ = 1		*ω*_1_ = 1					
	*ω*_2_ = 1		*ω*_2_ = 13.669					
*DvirGJ21443*	*p*_0_ = 0.587	−14,488.933	*p*_0_ = 0.682	−14,480.535	16.795	1	4.2 × 10^−5^	2.1 × 10^−4^
	*p*_1_ = 0.222		*p*_1_ = 0.256					
	*p*_2a_ = 0.139		*p*_2a_ = 0.045					
	*p*_2b_ = 0.052		*p*_2b_ = 0.017					
	*ω*_0_ = 0.143		*ω*_0_ = 0.144					
	*ω*_1_ = 1		*ω*_1_ = 1					
	*ω*_2_ = 1		*ω*_2_ = 15.825					
*DvirGJ22672/22673/26512*	*p*_0_ = 0.679	−14,491.793	*p*_0_ = 0.703	−14,488.424	6.739	1	9.4 × 10^−3^	1.7 × 10^−2^
	*p*_1_ = 0.260		*p*_1_ = 0.267					
	*p*_2a_ = 0.044		*p*_2a_ = 0.022					
	*p*_2b_ = 0.017		*p*_2b_ = 0.008					
	*ω*_0_ = 0.144		*ω*_0_ = 0.144					
	*ω*_1_ = 1		*ω*_1_ = 1					
	*ω*_2_ = 1		*ω*_2_ = 8.945					

### Statistical Analysis

The cumulative branch length (CBL) per clade was calculated by adding all branch lengths within a clade. Branch lengths were obtained as outputs of RAxML software (Stamatakis 2006). To take into account differences in number of sequences per clade, we calculated the normalized CBL, that is, the value of CBL/number of FAR sequences per clade. We compared the CBL means between stable and unstable FARs using a *t*-test as the CBL followed a normal distribution. Alternatively, we also calculated the cumulative patristic distance per clade by adding all branch lengths within a clade and the internal branch lengths from the root to the node supporting the clade. Statistical tests and graphics were performed using R statistics package version 3.5.0 (the R Project for Statistical Computing, www.r-project.org, last accessed April 27, 2018).

### Prediction of Putative Substrate Binding Sites

The putative substrate-binding sites of CG30427 proteins were predicted using the online tool CDD/SPARCLE (Marchler-Bauer et al. 2017). *CG30427* transcripts were used as queries to search for conserved and annotated coding sequences in NCBI’s Conserved Domain Database. The prediction relies on the 3D structure and highly conserved substrate-binding residues of some members of the extended Short-chain Dehydrogenase/Reductase family (Kavanagh et al. 2008).

### In Situ Hybridization in Embryos and Adult Oenocytes

In situ hybridization of oenocytes of embryos or 4–5-day-old adults was performed with RNA probes as described previously (Shirangi et al. 2009). Probes were made from mixed-sex 5-day-old adult cDNA using the primers listed in supplementary table S1, Supplementary Material online.

### RNAi Experiments

To determine if a given reductase was important for the viability of the fly, UAS-RNAi strains were individually crossed to tubulin-GAL4/TM3 *Sb*, resulting in RNAi knockdown in a ubiquitous pattern as previously described (Chung et al. 2009). Reciprocal crosses were performed at 25 °C. The sex and phenotype of emerging adults were scored. Stubble bristles were used to indicate the presence of the TM3, *Sb* chromosome in progeny, and therefore the absence of the tubulin-GAL4 chromosome. A specific reductase was scored as having an essential function if only flies carrying the TM3, *Sb* chromosome emerged from the cross (i.e., ubiquitous RNAi of the FAR resulted in lethality).

## Results

### Combined Phylogenetic and Microsynteny Analyses Identify Stable and Unstable Members of the FAR Gene Family in *Drosophila*

**Fig. 2.**
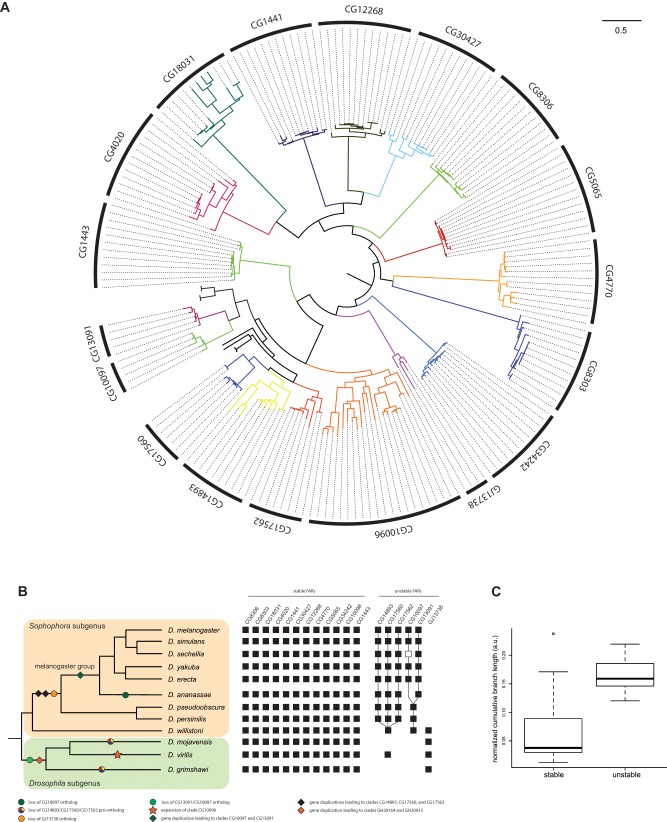
—Phylogeny and evolution of FAR genes in the *Drosophila* genus. (*A*) Phylogram of the 200-taxon analyses. RAxML maximum-likelihood analyses were conducted under the LG + Γ model. Support values are shown in supplementary fig. S1, Supplementary Material online. Scale bar indicates number of changes per site. (*B*) Molecular events underlying the origin and diversification of the FAR repertoire. Filled squares indicate the presence of a gene, and open squares indicate pseudogenes. (*C*) Comparison of CBLs between stable and unstable FAR clades.

To determine which members of the FAR gene family are evolutionarily stable or unstable, we employed a phylogenomic approach based on an unprecedented sampling of FAR sequences. We applied an exhaustive BLAST similarity search to the 12 available full *Drosophila* genomes. We found that the number of FARs in each of the 12 sequenced *Drosophila* genomes ranged from 14 to 21 (table 1). We then performed maximum-likelihood and Bayesian phylogenetic reconstruction on our 200-FAR data set (supplementary fig. S1, Supplementary Material online). The resulting tree clarifies the number of main FAR lineages within the *Drosophila* genus. The FAR sequences split into 18 main clades (fig. 2*A*). Out of these 18 clades, 3 clades originated through gene duplications specific to the *melanogaster* group such as the duplication leading to the clades *CG13091* and *CG10097*, and the one leading to the clades *CG17562*, *CG14893*, and *CG17560* (fig. 2*B*). After removal of these lineage-specific FAR clades, it is reasonable to infer that the last common ancestor of the extant *Drosophila* genus possessed at least 15 FAR genes.

**Table 1:** FAR Content Gene in the 12 Sequenced *Drosophila* Genomes

Species	FAR Content	Genome	Previous Estimates
*Drosophila melanogaster*	17	v. 6.06	13 (Lassance et al. 2010); 15 (Eirin-Lopez et al. 2012)
*Drosophila simulans*	17	v. 2.01	This study
*Drosophila sechellia*	17	v. 1.3	This study
*Drosophila yakuba*	17	v. 1.04	This study
*Drosophila erecta*	17	v. 1.04	This study
*Drosophila ananassae*	17	v. 1.04	This study
*Drosophila pseudoobscura*	21	v. 3.03	This study
*Drosophila persimilis*	20	v. 1.3	This study
*Drosophila willistoni*	15	v. 1.04	This study
*Drosophila mojavensis*	14	v. 1.04	This study
*Drosophila virilis*	20	v. 1.03	This study
*Drosophila grimshawi*	14	v. 1.3	This study

Note.—The number of FARs ranges from 14 to 21, with 17 genes in the model species *D. melanogaster*.

Our inference of the number of clades is also well supported by the study of microsynteny conservation between species. Each clade includes orthologous sequences whose relative genomic location is conserved between the 12 *Drosophila* genomes (supplementary fig. S2, Supplementary Material online). We identified a set of 12 stable FARs with at least one copy in all 12 genomes (fig. 2). Conversely, we identified a set of unstable FAR clades that show variable gene content among *Drosophila* species. Moreover, unstable FAR and stable FAR genes have very distinct branch lengths. We found longer branch lengths for unstable FARs using cumulative clade branch length (*t*-test: df = 13, *P* = 0.003; fig. 2*C*) or cumulative patristic distance (*t*-test: df = 5, *P* = 0.01; supplementary fig. S3, Supplementary Material online), suggesting that unstable FAR genes evolve faster than stable FAR genes.

### The *Drosophila* FAR Repertoire Evolved through Multiple Gene Duplication Events and Independent Gene Losses

Several independent gene losses have occurred during the evolution of the FAR gene family. For example, FAR genes of the clade *CG13091*/*CG10097* are absent from the entire subgenus *Drosophila* (*grimshawi*, *mojavensis*, and *virilis* groups), and the *CG10097* ortholog is absent from *D. ananassae* (fig. 2*B*). Moreover, the *CG10097* ortholog in *D. sechellia* (*DsecGM26015*) shows clear features of pseudogenization such as a fast evolution rate (supplementary fig. S1, Supplementary Material online and table 2) and a 16-bp deletion that results in a truncated putative transcript. In contrast, genes of the clade *CG13091*/*CG10097* have been duplicated and retained in most species of the subgenus *Sophophora* (except for the *ananassae* group).

Another case of FAR gene loss is in the clade *GJ13738*, which is restricted to the entire subgenus *Drosophila* and the *willistoni* group. When mapped onto the *Drosophila* species tree, the distribution of the clade *GJ13738* suggests a unique loss event in the subgenus Sophophora after the divergence of the *willistoni* group (fig. 2*B*). A third example of FAR gene loss is in the ancestral clade *CG14893*/*CG17560*/*CG17562* which is restricted to the subgenus *Sophophora* and *D. virilis*. We find two independent losses of this clade in the lineage leading to *D. mojavensis* and *D. grimshawi*, respectively (fig. 2*B*). Conversely, this clade went through three successive rounds of gene duplications within the *Sophophora* subgenus.

### Signatures of Positive Selection Associated with Repeated Duplication Events

The most striking example of FAR content expansion is of the *CG10096* orthologs in *D. virilis* (fig. 2*A* and *B*). Eight copies have been identified in the genome of *D. virilis*. This specific expansion contributes to a higher FAR content in *D. virilis* (table 1), as well as an increase of the CBL for the *CG10096* clade (see the outlier dot, fig. 2*C*). This latter observation could result from a faster rate of molecular evolution due to relaxed selective pressure. We tested this hypothesis by searching for any signatures of positive selection in the *CG10096* clade. We did detect several branches and sequences of *D. virilis* under positive selection (table 2). We also noted expansion of the *CG14893* clade in *D. ananassae* to three copies. We detect signatures of positive selection in two of these paralogs (table 2).

### Evolution of Putative New Substrate Specificity: The Unique Case of Clade *CG30427*

**Fig. 3.**
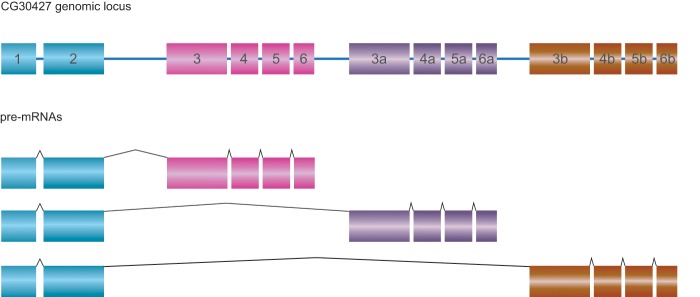
—Genomic structure and isoforms of the gene *CG30427* in *Drosophila melanogaster*. Exons 3–6 underwent duplication in tandem and do exist in three different copies. The combination of the three different “cassettes” with the single exons 1–2 leads to three different isoforms.

We have shown that gene duplication has played a major role in the diversification of the FAR family. We also observed an interesting case of alternative splicing affecting FAR diversity in the CG30427 clade. In *D. melanogaster*, the gene *CG30427* produces three main classes of transcripts (fig. 3). Surprisingly, the *CG30427* transcript variants have a highly conserved exon/intron structure and encode similar protein isoforms. These observations suggest that the gene *CG30427* could have evolved by serial duplication of exons 3–6 leading to repetition of the structural domains. Independent evolution (e.g., mutation) of the repeated exons, as well as the establishment of alternative splicing, could have subsequently generated distinct, but still comparable, isoforms. Notably, one of the predicted substrate-binding sites shows an amino acid difference between isoforms A and C (**M**ethionine) and isoform B (**V**aline) (supplementary fig. S4, Supplementary Material online). This may be significant because in *Arabidopsis thaliana*, the two enzymes FAR5 and FAR8 are 85% identical at the amino acid level, but they possess distinct substrate specificities for 18:0 or 16:0 acyl chain lengths, respectively (Chacón et al. 2013). Moreover, it has been recently shown that just two individual amino acid substitutions (L355A and M377V) explain most of the difference in substrate specificity between FAR5 and FAR8 (Chacón et al. 2013). Although there is no direct biochemical evidence available yet, we infer that the *D. melanogaster CG30427* transcript variants probably encode functionally distinct isoforms.

### Unstable FARs Are Mostly Expressed in the Oenocytes, the Site of CHC Biosynthesis

**Fig. 4.**
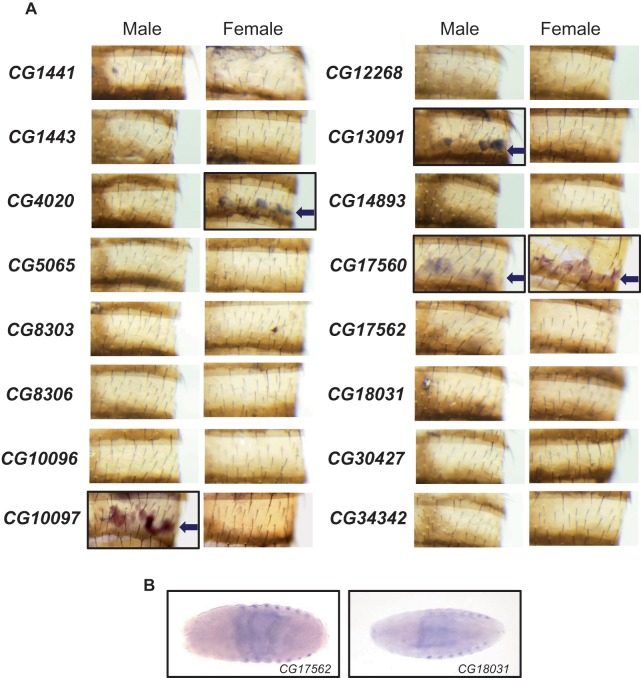
—Expression of FARs in *Drosophila melanogaster* adult cuticle and embryos. (*A*) *In situ* hybridizations of FARs in abdominal cuticle show that only four FARs are expressed in adult oenocytes (see arrows). (*B*) *In situ* hybridizations of the two FARs *CG17562* and *CG18031* that are expressed in embryonic oenocytes.

The biosynthesis of fatty acyl-CoA takes place in many tissues in the fly (Jaspers et al. 2014), but the biosynthesis of CHCs specifically occurs in the oenocytes (Billeter et al. 2009; Wicker-Thomas et al. 2015) (fig. 1). To determine which FARs may be involved in CHC production, we identified FARs expressed in the oenocytes by *in situ* hybridization in *D. melanogaster*. Using DIG-labeled RNA probes for 16 FARs (all except *CG4770*), we performed in situ hybridization on both mixed stage embryos and dissected adult abdomens. We detected expression of four FARs in oenocytes from adults. Three of these are evolutionarily unstable: *CG13091* (male expressed), *CG10097* (male expressed), and *CG17560* (expressed in both sexes). The only evolutionarily stable FAR expressed in oenocytes is *CG4020* (female expressed) (fig. 4). *In situ* hybridization in embryos showed only *CG17562* and *CG18031* (*FarO*) are expressed in embryonic oenocytes (fig. 4), whereas the other FARs are expressed in other tissues such as the salivary glands and the tracheal system (supplementary fig. S5, Supplementary Material online). No FAR is expressed in both embryonic and adult oenocytes.

### Stable FARs Are Likely to Have Essential Functions

To determine if the loss of a FAR impacts viability, we used the ubiquitous tubulin-GAL4 driver and UAS-RNA interference (RNAi) to knock down each FAR individually. We found that RNAi knockdown of 9 out of the 12 stable FARs (75%) led to mortality while only 1 out of 5 unstable FARs (20%) was essential for viability (table 3). These results demonstrate that the majority of stable FARs are essential for viability and confirm previous work on two specific FARs including the *CG1443* gene (*wat*), which is expressed in the trachea and involved in gas filling of the tracheal tubes during *Drosophila* embryogenesis (Jaspers et al. 2014), and the *CG18031* gene (*FarO*), which plays a key role in preventing excessive oenocyte cell growth (Cinnamon et al. 2016). In contrast, we deduce that most of the unstable FARs are involved in nonessential functions that evolve rapidly between species.

**Table 3:** RNAi Knockdown of Individual FAR Genes Using the Ubiquitous Tubulin-GAL4 Driver

Phylogenetic Stability	Gene Name	RNAi Phenotype	Adult Oenocyte Expression
Stable	*CG1443*	*Lethal*	N
	*CG4020*	*Lethal*	*Yes (female)*
	*CG4770*	*Lethal*	N
	*CG5065*	*Lethal*	N
	*CG8303*	*Lethal*	N
	*CG8306*	*Lethal*	N
	*CG10096*	*Lethal*	N
	*CG12268*	*Lethal*	N
	*CG34342*	*Lethal*	N
	*CG1441*	Viable	N
	*CG18031*	Viable	N
	*CG30427*	Viable	N
Unstable	*CG10097*	Viable	*Yes (male)*
	*CG13091*	Viable	*Yes (male)*
	*CG14893*	Viable	N
	*CG17562*	Viable	N
	*CG17560*	*Lethal*	*Yes (both)*

Note.—Most of evolutionary stable members of this gene family are essential for development (lethal when knocked down by RNAi), whereas most of the evolutionary unstable members of this gene family are involved in nondevelopmental processes (viable when knocked down by RNAi).

## Discussion

Using a combination of bioinformatics and reverse genetics, we have conducted a comprehensive study of the FAR gene family in the genus *Drosophila*. We have shown that 5 out of 17 FARs found in the *D. melanogaster* genome are evolutionary unstable. Most of these unstable FARs are expressed in the oenocytes, the site of CHC biosynthesis in *D. melanogaster*, compared with only 1 of the 12 stable FARs. Our functional RNAi experiments demonstrate that most stable FARs carry out have functions crucial for viability, whereas silencing most unstable FARs do not lead to lethality. These data suggest that the gain and loss of unstable FARs can alter CHC diversity without affecting insect viability, although the effects on organismal fitness are unclear. Comparison of CBLs between stable and unstable FAR clades showed that unstable FARs undergo more rapid sequence evolution compared with stable FARs. Taken together, we suggest that FAR genes involved in CHC synthesis are likely to be evolutionary unstable and evolve faster than other FARs. These results appear to support an important but largely untested tenet of the birth-and-death model of gene families (Nei and Rooney 2005; Eirin-Lopez et al. 2012)—that stable members of gene families often encode genes with core functions involved in viability, whereas unstable members often encode genes involved in nonviable and rapidly evolving functions (Thomas 2007).

However, the birth-and-death of fatty acyl-CoA biosynthesis gene family members is not the only mechanism underlying the rapid divergence of CHCs between species. The fatty acyl-coA desaturase (desat) gene family is another gene family involved in the synthesis of CHCs. Three desats (*desat1*, *desat2* and *desatF*) have been experimentally shown to be involved in CHC synthesis in *Drosophila* (Dallerac et al. 2000; Takahashi et al. 2001; Chertemps et al. 2006). *desat1* is an evolutionary stable gene which has pleiotropic functions in *D. melanogaster* (Bousquet et al. 2012), whereas *desat2* was lost in *D. erecta*, and the *desatF* lineage went through several rounds of gene duplication and subsequent specific gene losses (Fang et al. 2009; Keays et al. 2011). Regulatory changes that affect oenocyte expression, as well as transition from monomorphic to dimorphic oenocyte expression (and its reversion) of *desatF*, account for CHC divergence caused by this gene as well (Shirangi et al. 2009). *Cis*-regulatory changes in other fatty acyl-CoA biosynthesis genes have also been shown to be involved in CHC divergence between *Drosophila* species. These include *cis*-regulatory changes in *mFAS* (a fatty acid synthase) expression between two closely related Australian *Drosophila* species (Chung et al. 2014), as well as a recent discovery that tissue-specific *cis*-regulatory changes affect the expression of *eloF*, a fatty acid elongase, leading to CHC divergence and mating inhibition in *D. simulans* and *D. sechellia* (Combs et al. 2018). Based on the evidence obtained to date, we suggest that the birth-and-death of fatty acyl-CoA biosynthesis genes, as well as *cis*-regulatory evolution, accounts for the majority of CHC evolution in *Drosophila*. We note that no example of coding changes has been shown thus far to account for CHC divergence.

Differences in gene family content allow the diversification and ecological adaptation of many different species (Demuth and Hahn 2009; Żmieńko et al. 2014; Carretero-Paulet et al. 2015). The advancement of sequencing technologies has led to sequencing and availability of more than 100 insect genomes (Yin et al. 2016) with a few thousand more being proposed (i5K Consortium 2013). This includes closely related species such as 16 *Anopheles* mosquito genomes (Neafsey et al. 2015). The birth-and-death evolution model could be used to identify genes involved in rapidly evolving traits between species such as CHC synthesis. Because CHCs are involved in premating isolation between many closely related insect species, identification of evolutionarily unstable genes may also shed light on the speciation and radiation of such groups.

## Data Accessibility

New sequence of the *D. yakuba* transcript *FarO* (*DyakGE28152*) was deposited in the EMBL database (accession number LT996250). Sequence alignment and tree files are downloadable from Dryad (doi:10.5061/dryad.s31rc70).

## Supplementary Material

Supplementary data are available at *Genome Biology and Evolution* online.

## Supplementary Material

Supplementary_Material_evz094

## Notes

**Data deposition:** New sequence of the *Drosophila yakuba* transcript FarO (DyakGE28152) was deposited at the EMBL database under the accession LT996250. Sequence alignment and tree files are downloadable from Dryad (doi:10.5061/dryad.s31rc70).
