# Supplementary material for: Birth-and-Death Evolution of the Fatty Acyl-CoA Reductase (FAR) Gene Family and Diversification of Cuticular Hydrocarbon Synthesis in Drosophila
Source: Genome Biol Evol. 2019 May 10;11(6):1541–51. doi: 10.1093/gbe/evz094 (PMC6546124; doi:10.1093/gbe/evz094)
Supplement: Supplementary_Material_evz094 [file supplementary_material_evz094.zip › Finet et al. Suppl Materials 032019.pdf]

Fig S1. Bayes tree

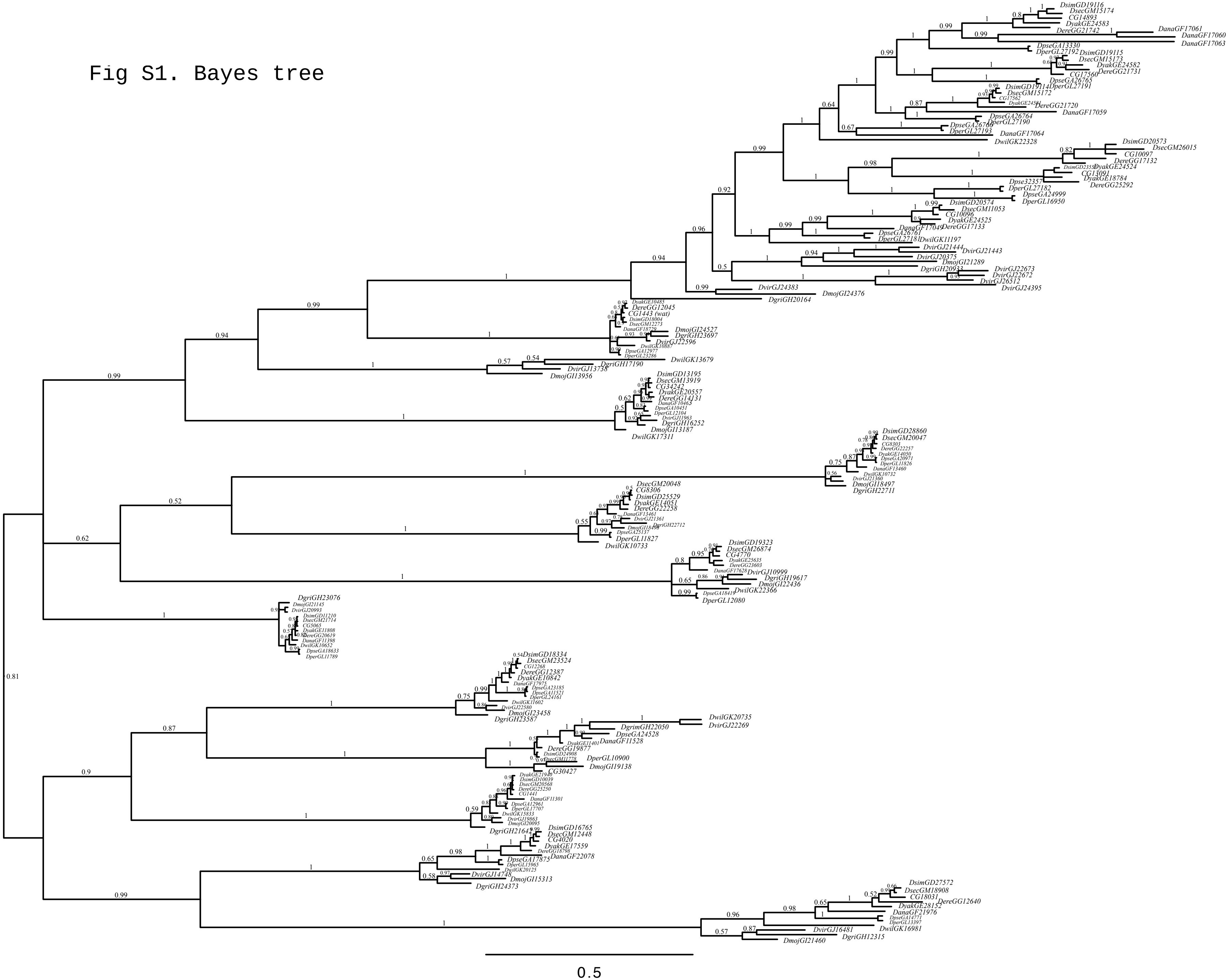

Fig S1. ML tree

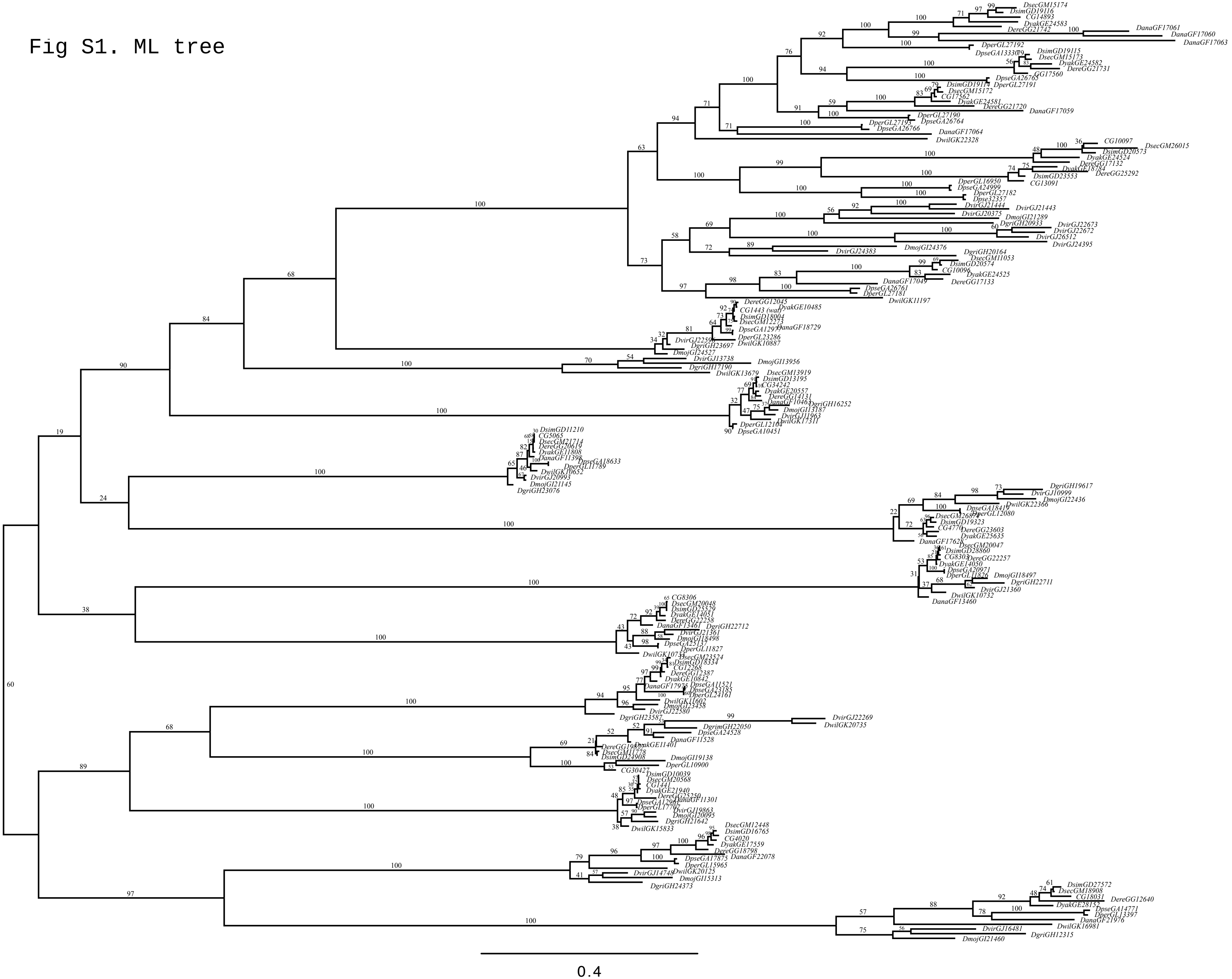

Fig. S2

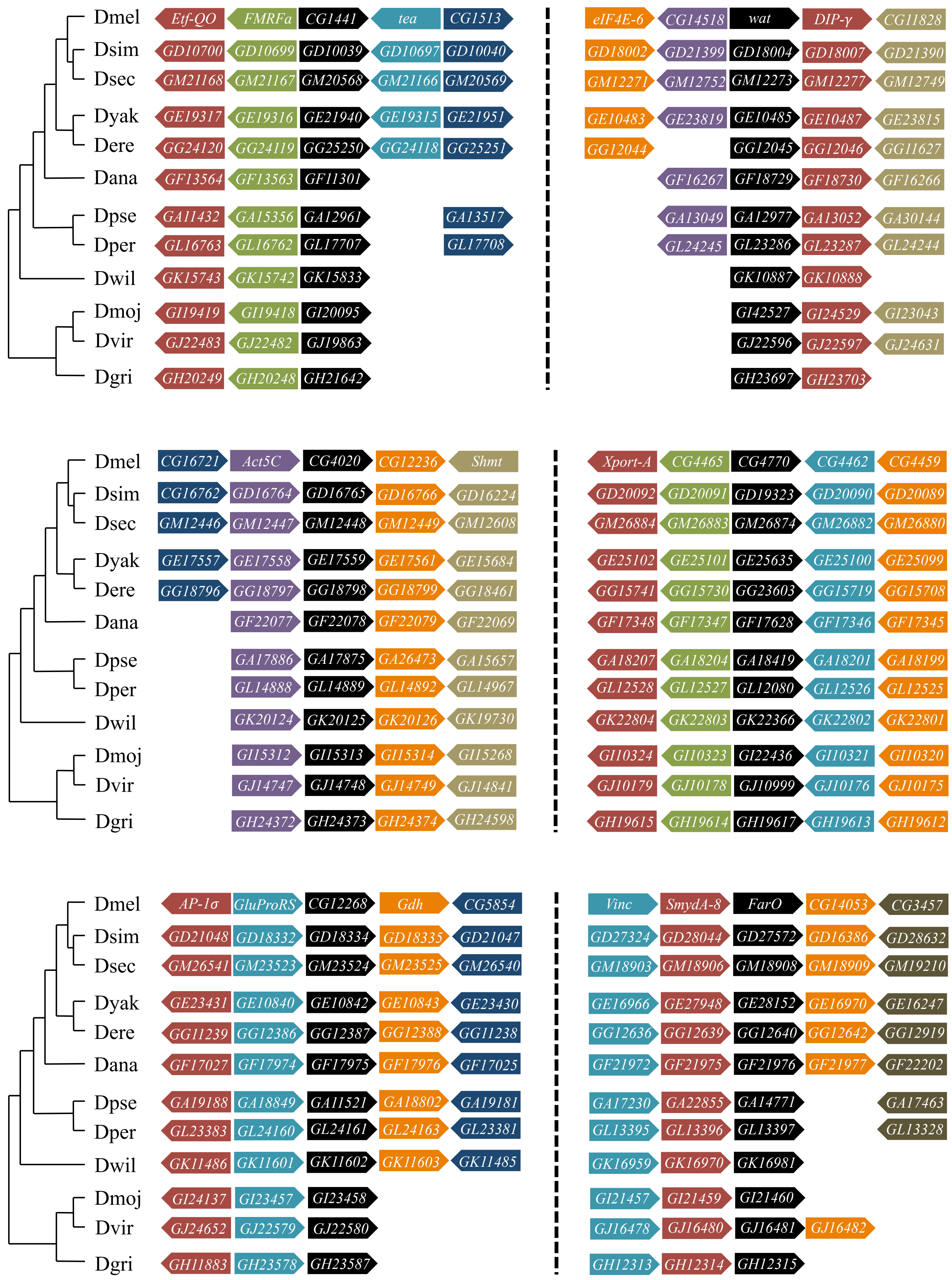
Dmel Etf-QO FMRFa CG1441 tea CG1513 eIF4E-6 CG14518 wat DIP-γ CG11828
Dsim GD10700 GD10699 GD10039 GD10697 GD10040 GD18002 GD21399 GD18004 GD18007 GD21390
Dsec GM21168 GM21167 GM20568 GM21166 GM20569 GM12271 GM12752 GM12273 GM12277 GM12749
Dyak GE19317 GE19316 GE21940 GE19315 GE21951 GE10483 GE23819 GE10485 GE10487 GE23815
Dere GG24120 GG24119 GG25250 GG24118 GG25251 GG12044 GG12045 GG12046 GG11627
Dana GF13564 GF13563 GF11301 GF16267 GF18729 GF18730 GF16266
Dpse GA11432 GA15356 GA12961 GA13517 GA13049 GA12977 GA13052 GA30144
Dper GL16763 GL16762 GL17707 GL17708 GL24245 GL23286 GL23287 GL24244
Dwil GK15743 GK15742 GK15833 GK10887 GK10888
Dmoj GI19419 GI19418 GI20095 GI42527 GI24529 GI23043
Dvir GJ22483 GJ22482 GJ19863 GJ22596 GJ22597 GJ24631
Dgri GH20249 GH20248 GH21642 GH23697 GH23703
Dmel CG16721 Act5C CG4020 CG12236 Shmt Xport-A CG4465 CG4770 CG4462 CG4459
Dsim CG16762 GD16764 GD16765 GD16766 GD16224 GD20092 GD20091 GD19323 GD20090 GD20089
Dsec GM12446 GM12447 GM12448 GM12449 GM12608 GM26884 GM26883 GM26874 GM26882 GM26880
Dyak GE17557 GE17558 GE17559 GE17561 GE15684 GE25102 GE25101 GE25635 GE25100 GE25099
Dere GG18796 GG18797 GG18798 GG18799 GG18461 GG15741 GG15730 GG23603 GG15719 GG15708
Dana GF22077 GF22078 GF22079 GF22069 GF17348 GF17347 GF17628 GF17346 GF17345
Dpse GA17886 GA17875 GA26473 GA15657 GA18207 GA18204 GA18419 GA18201 GA18199
Dper GL14888 GL14889 GL14892 GL14967 GL12528 GL12527 GL12080 GL12526 GL12525
Dwil GK20124 GK20125 GK20126 GK19730 GK22804 GK22803 GK22366 GK22802 GK22801
Dmoj GI15312 GI15313 GI15314 GI15268 GI10324 GI10323 GI22436 GI10321 GI10320
Dvir GJ14747 GJ14748 GJ14749 GJ14841 GJ10179 GJ10178 GJ10999 GJ10176 GJ10175
Dgri GH24372 GH24373 GH24374 GH24598 GH19615 GH19614 GH19617 GH19613 GH19612
Dmel AP-1σ GluProRS CG12268 Gdh CG5854 Vinc SmydA-8 FarO CG14053 CG3457
Dsim GD21048 GD18332 GD18334 GD18335 GD21047 GD27324 GD28044 GD27572 GD16386 GD28632
Dsec GM26541 GM23523 GM23524 GM23525 GM26540 GM18903 GM18906 GM18908 GM18909 GM19210
Dyak GE23431 GE10840 GE10842 GE10843 GE23430 GE16966 GE27948 GE28152 GE16970 GE16247
Dere GG11239 GG12386 GG12387 GG12388 GG11238 GG12636 GG12639 GG12640 GG12642 GG12919
Dana GF17027 GF17974 GF17975 GF17976 GF17025 GF21972 GF21975 GF21976 GF21977 GF22202
Dpse GA19188 GA18849 GA11521 GA18802 GA19181 GA17230 GA22855 GA14771 GA17463
Dper GL23383 GL24160 GL24161 GL24163 GL23381 GL13395 GL13396 GL13397 GL13328
Dwil GK11486 GK11601 GK11602 GK11603 GK11485 GK16959 GK16970 GK16981
Dmoj GI24137 GI23457 GI23458 GI21457 GI21459 GI21460
Dvir GJ24652 GJ22579 GJ22580 GJ16478 GJ16480 GJ16481 GJ16482
Dgri GH11883 GH23578 GH23587 GH12313 GH12314 GH12315

Fig. S2

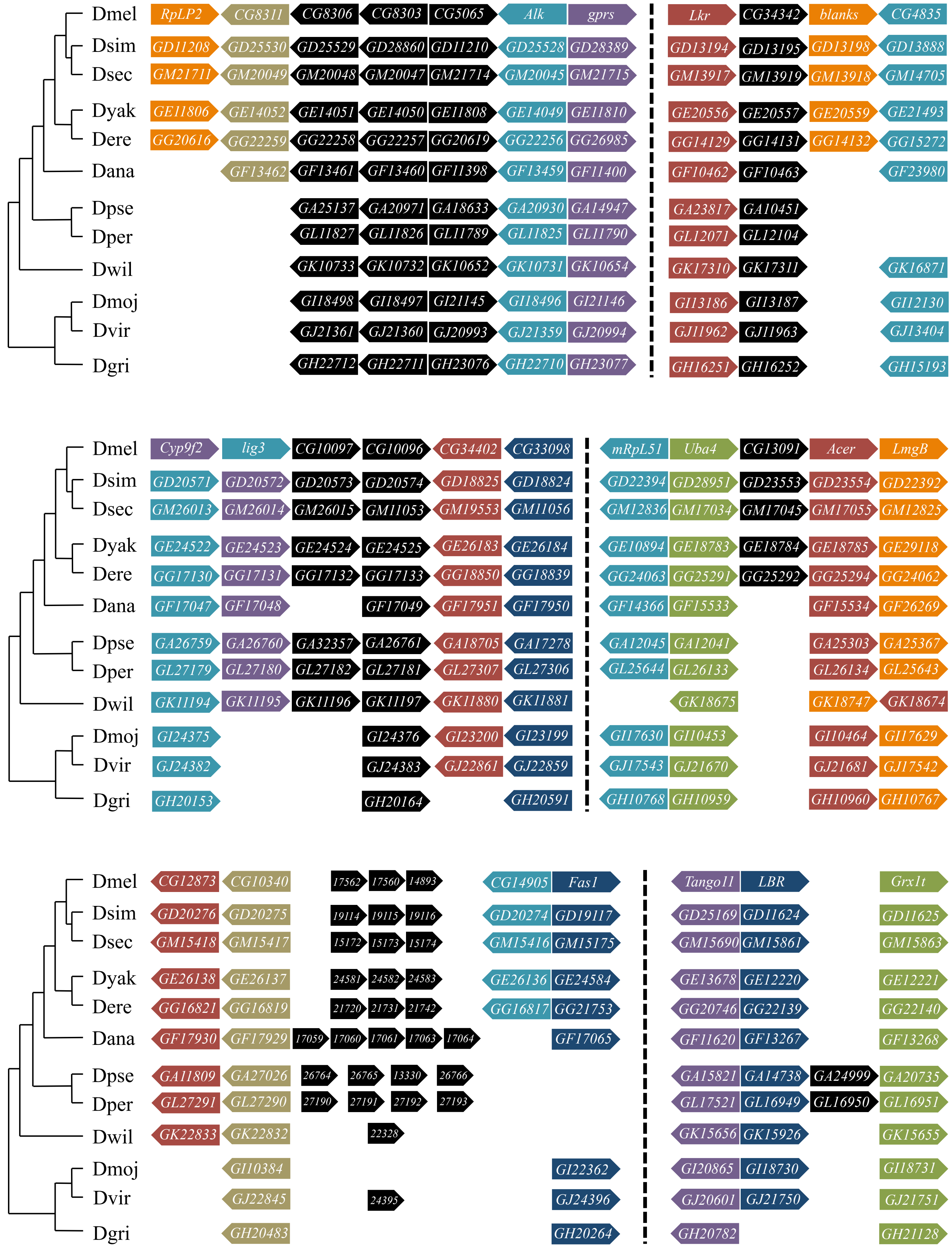
Dmel
Dsim
Dsec
Dyak
Dere
Dana
Dpse
Dper
Dwil
Dmoj
Dvir
Dgri
RpLP2
CG8311
CG8306
CG8303
CG5065
Alk
gprs
Lkr
CG34342
blanks
CG4835
Cyp9f2
lig3
CG10097
CG10096
CG34402
CG33098
mRpL51
Uba4
CG13091
Acer
LmgB
CG12873
CG10340
CG14905
Fas1
Tango11
LBR
Grx1t

Fig S3

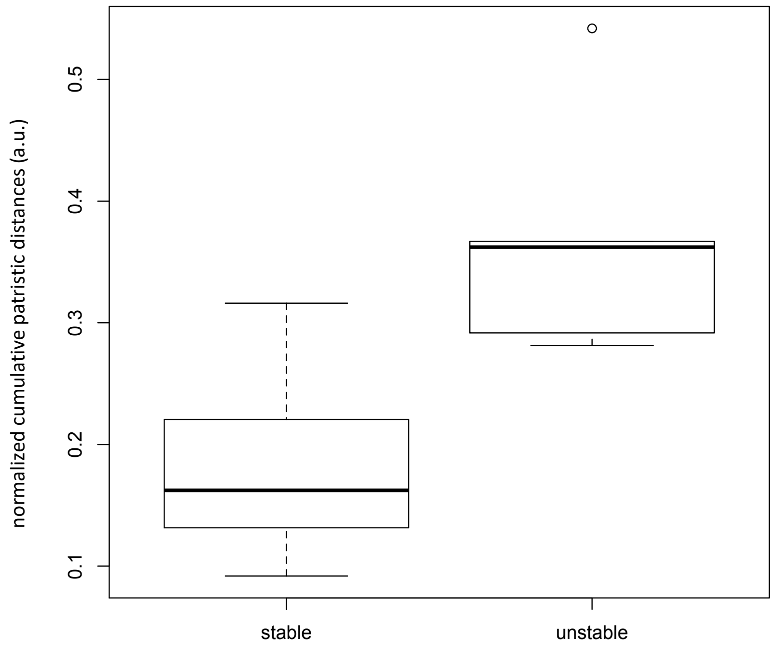

normalized cumulative patristic distances (a.u.)
0.1
0.2
0.3
0.4
0.5
stable
unstable

Fig. S4

```
CLUSTAL O(1.2.1) multiple sequence alignment


protC      MTVDLSPVQEYYKDKTIFITGASGFMGKVLLEKLLYSCHSLKEVIIICRPKRGKAPETRL
protA      MTVDLSPVQEYYKDKTIFITGASGFMGKVLLEKLLYSCHSLKEVIIICRPKRGKAPETRL
protB      MTVDLSPVQEYYKDKTIFITGASGFMGKVLLEKLLYSCHSLKEVIIICRPKRGKAPETRL
           ************************************************************

protC      EEMFKLPIFQRIKDERPEMLKRVTIYQGDVTFDQLGLSGESLKHVTENTNIVFHMAATLK
protA      EEMFKLPIFQRIKDERPEMLKRVTIYQGDVTFDQLGLSGESLKHVTENTNIVFHMAATLK
protB      EEMFKLPIFQRIKDERPEMLKRVTIYQGDVTFDQLGLSGESLKHVTENTNIVFHMAATLK
           ************************************************************

protC      LEGNLRDAIDMNLLGTRRALNVAKEMKQLEAFIHLSTAFCNCDQDVMYEKVYEFPHKPED
protA      LEGNLRDAIDMNLLGTRRALNVAKEMKQLEAFIHLSTAFCNCDQDVMYEKVYEFPHKPED
protB      LEGNLRDAIDMNLLGTRRALNVAKEMKQLEAFIHLSTAFCNCDQDVMYEKVYEFPHKPED
           ************************************************************

protC      LMRLAEWMDVKTLDAITPDLLKPHPNTYTYSKRLAELLVRDHYESMPVIIARPSIVTPAV     Beginning
protA      LMRLAEWMDVKTLDAITPDLLKPHPNTYTYSKRLAELLVRDHYESMPVIIARPSIVSPSA      exon 3
protB      LMRLAEWMDVKTLDAITPDLLKPHPNTYTYSKRLAELLVRDHYESMPVIIARPSIVSPAA
           ********************************************************:*:.

protC      AEPLPGWVDNMNGPTGVLIGAGKGVIRSMICNGELKSEVIPVDIAINGLILLPYHNSLAE
protA      YEPVPGWVDNLNGPTGLMVGAGKGVIRSMLIDTRHLSEVIPVDYAINGLCVIPYQFAKMT
protB      YEPLPGWVDNLNGPTGLMIGCGKGVIRSVLVNQQNKAEVIPVDYAINGLIVIPYEFNKQA
            **:******:*****:::*.*******:: : .  :****** ***** ::**.

protC      KRPLQIPVYNLTVDDAKKRTWKWIMDVGRDLGIKYPFEVGLWYPDGNMTSSKFYHTICTI
protA      ERPKDVPVYNITCADHRKMQWGEVIEMSKEIGYRYPMEAGLWYPDGCITTNKLHHNINVL
protB      KRPTNVPVYNITNADHRKMTMGTVVEMSKRINKQFPFNAGLWYPDPCVTTNQLYHNFNVA
           :** ::****:*  * :*     ::::.: :  ::*::.******  :*:.:::*.: .

protC      LFMWLPAYLIDFLLLIFGQRRFMIRVQTKIAVGLEVLQFFTTRSWDFKSTHFEQIYKELG
protA      LFHWLPAYLIDFILLLLGQKRFMIRVQNRISVGLEVLQFFTMRAWFFKSDAYSSLWAMLN
protB      LFHWLPAYFLDFLMLILGQKRFMLRVQEKISTGLGVLQFFTLNAWCFRSDNYASLWNKLN
           ** *****::**::*::**:***:*** :*:.** ****** .:* *:*  : .::  *

protC      STDRRIFKINTDDVD-DYEYMKVSILGGRQYVMKEPLTSLPKSRIQLRFMYVLDRICKTM
protA      ESDRKNFNMDMDPEETVPMYIESCVQGGRQYLMKESPDSLPRARLQLKLMYILDRACKTL
protB      EEDKAIFNMNMNTENTEEEYMIECAKGARKFILKEKEEDLPTARVHMKIQRVVDVVCKTV
           . *:  *::: :  :    *:  .  *.*::::**   .** :*:::::  ::*  ***:

protC      IISGLLYWVSQKLRVVDLFRSVFHSST
protA      IVGTLCYWTYGLVARLLGV--------
protB      IVGLFFYYLLKWTGVLAIF--------
           *:. : *:       :  .
```

Fig. S5

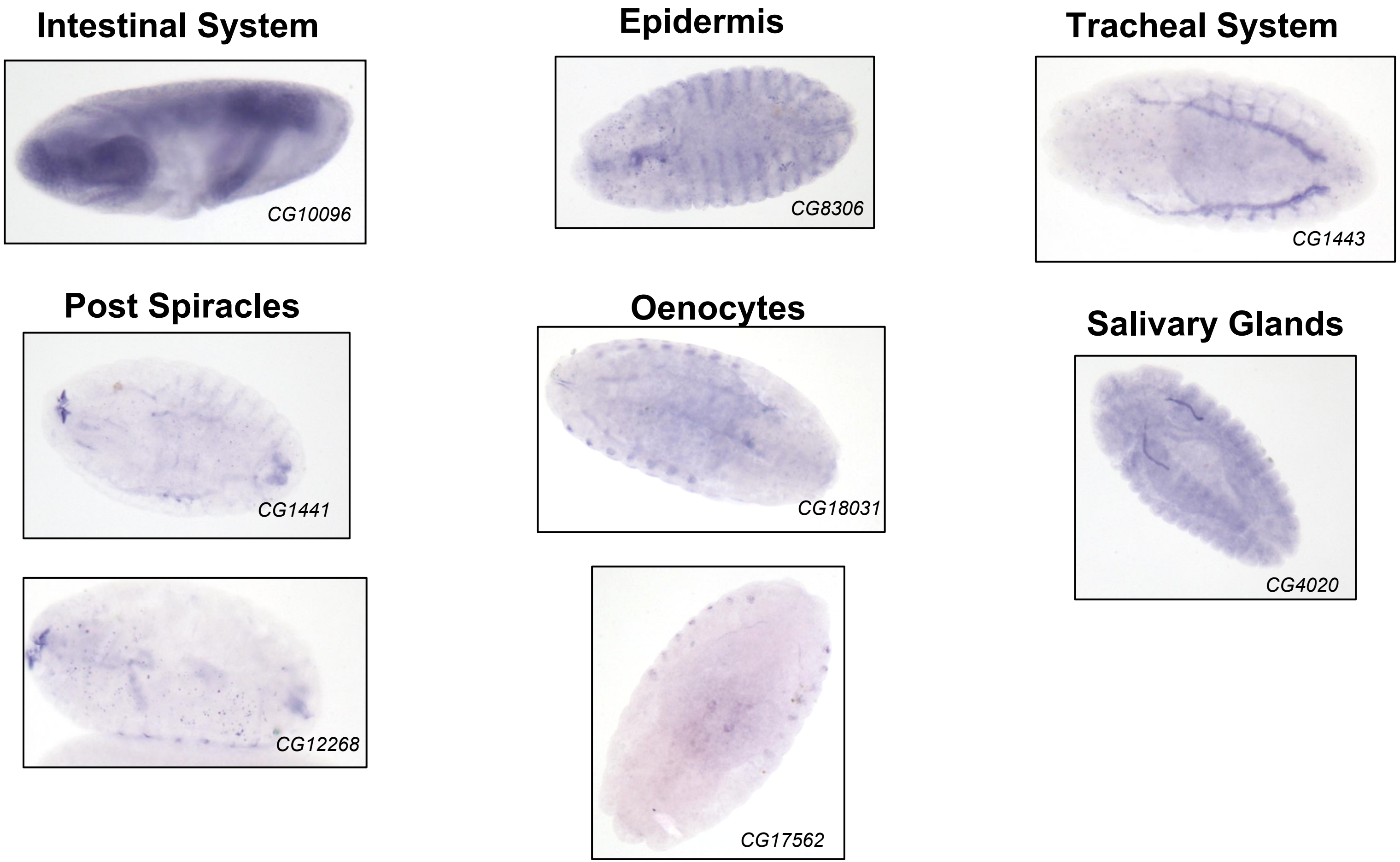
Intestinal System
CG10096
Epidermis
CG8306
Tracheal System
CG1443
Post Spiracles
CG1441
CG12268
Oenocytes
CG18031
CG17562
Salivary Glands
CG4020

Primers	Sequence 5'-3'
CG1441probeF	TTCGATGAAGTCCAACTATTG
CG1441probeR	AGAGCACACAGTTGGTGAAG
CG1443probe-F	ATACGAGACTCCGCCGATTTAC
CG1443probe-R	ATAAGAATAGCTTGAGCAGGGC
DmCG4020-ex4F	GTATGGTAACTTTCAGCCGAACAC
DmCG4020-ex4R	CCGCACAAAATGAAGAACCGGAC
CG4770probe-F	CTTCAAGTATAGGTGCAGTGC
CG4770probe-R	CTAAAATGAAATATAGACAAGC
CG5065probe-F	AGGGCTGCCTGTGGTATCCGAC
CG5065probe-R	TTACAAAAAGGGTATCAAGCGCG
CG8303probe-F	GAATCGGAATGCCCCAGCACAC
CG8303probe-R	CTACAACCAGACGAGAAATCCCCAG
CG8306probeF	TGCGTCTGGTGAAGAGCCT
CG8306probeR	CGAGGATCGTATTGGAAAAGT
CG10096probe-F	TGCGTTTATGGGGCAAAAAGC
CG10096probe-R	TTATACAAATGAGCCCAGTAGTCGC
CG10097probe-F	TGTATAACATTGGTATCTTATTC
CG10097probe-R	TTACAGTAGTGGCACTAAATTTG
CG12268probe-F	ACTACCACCACCTTTTCTGCGTC
CG12268probe-R	ATACATGTATGGTGCTGTTTCGC
DmCG13091-ex4F	TTTTGGCAAATTTCAAGGAGCCG
DmCG13091-ex4R	CTGAATAAACGCTTTTTCGCTTG
DmelCG14893-probeF	TTTTCCCCTTAGCCGCCTTCTTCT
DmelCG14893-probeR	AGGGCCTTCCGGAAGTACTCTTTCCA
DmelCG17560-probeF	TATTTCCGCTGGCCGCCTTCTTCT
DmelCG17560-probeR	ATGGCGGCCTTGAAATAGTCATTCCA
DmelCG17562-probeF	TCTTCCCCCTGGCAGCTTTCTTCT
DmelCG17562-probeR	AGAGCTTGAACGAAGTATTCCTTCCA
CG18031probeF	GCAAGGCCTACCAAACGCT
CG18031probeR	TTCTCCAAAGCCAGAGGATC
CG30427-RCprobeF	AACATGACGTCCAGCAAATTC
CG30427-RCprobeR	CCACCCAAAATACTGACTTTCA
CG34342probe-F	ATGCACGAGGCAAATCCAA
CG34342probe-R	CGATTCATTGAAAAAGCCCTTC

Gene name	VDRC-ID	ON targets	Off targets
CG1441	GD4034	1	1 (CG14162)
CG1443	KK105518	1	0
CG4020	KK107095	1	0
CG4770	KK101641	1	0
CG5065	KK108205	1	0
CG8303	KK103744	1	0
CG8306	KK113348	1	0
CG10096	GD50765	2 (CG10097)	1
CG10097	GD6089	2 (CG10096)	1 (CG16974)
CG12268	GD1166	1	0
CG13091	KK106304	1	0
CG14893	KK100324	1	0
CG17560	KK104756	1	2 (CG10096/CG10097)
CG17562	GD37365	1	2 (CG10096/CG10097)
CG18031	KK107564	1	1 (CG31019)
CG30427	KK102330	1	0
CG34342	KK104554	1	2 (CG12109, CG10936)
