# Supplementary material for: Birth-and-Death Evolution of the Fatty Acyl-CoA Reductase (FAR) Gene Family and Diversification of Cuticular Hydrocarbon Synthesis in Drosophila
Source: Genome Biol Evol. 2019 May 10;11(6):1541–51. doi: 10.1093/gbe/evz094 (PMC6546124; doi:10.1093/gbe/evz094)
Supplement: Supplementary_Material_evz094 [file supplementary_material_evz094.zip › Supplementary data.docx]

**Supplementary data**

**Fig. S1.** Phylograms of the 200-taxon analyses. RAxML maximum-likelihood analyses and PhyloBayes Bayesian analyses were conducted under the LG and the GTR+Γ model, respectively. Support values obtained after 100 bootstrap replicates and Bayesian posterior probabilities are show for all branches. Scale bar indicates number of changes per site.

**Fig. S2.** Synteny conservation at FAR genomic loci and adjacent genes in the *Drosophila* genus.

**Fig. S3.** Unstable FARs evolve faster than stable FARs. The plot shows the normalized cumulative branch length (using patristic distances) for stable and unstable FARs.

**Fig. S4.** Alignment and divergence of proteins encoded by the gene *CG30427*. Putative substrate binding sites are underlined in red. The single substitution among isoforms that affects a putative binding site is highlighted in yellow.

**Fig. S5.** Expression of some of the FARs in *D. melanogaster* embryos.

**Table S1.** List and sequence of primers used in this study.

**Table S2**. Fly RNAi lines used in this study.
